# Daunorubicin can eliminate iPS-derived cancer stem cells via ICAD/CAD-independent DNA fragmentation

**DOI:** 10.20517/cdr.2019.01

**Published:** 2019-06-19

**Authors:** Akimasa Seno, Akifumi Mizutani, Kazuki Aizawa, Ryoma Onoue, Junko Masuda, Naotaka Ochi, Saki Taniguchi, Tatsuyuki Sota, Yuki Hiramoto, Taisuke Michiue, Neha Nair, Masaharu Seno

**Affiliations:** ^1^Laboratory of Nano-Biotechnology, Graduate School of Interdisciplinary Science and Engineering in Health Systems, Okayama University, Okayama 700-8530, Japan.; ^2^Division of Medical Bioengineering, Graduate School of Natural Science and Technology, Okayama University, Okayama 700-8530, Japan.

**Keywords:** Daunorubicin, cancer stem cell, DNA fragmentation

## Abstract

**Aim:** To identify a drug that can effectively eliminate these cancer stem cells (CSCs) and determine its mode of action.

**Methods:** CSCs were obtained from mouse induced pluripotent stem cells (miPSCs) using cancer cell-conditioned media. Drug screening was performed on these cells or after transplantation into mice. Apoptosis was analyzed by flow cytometry and western blotting.

**Results:** Drug screening studies showed that daunorubicin, a topoisomerase II inhibitor, is specifically cytotoxic to miPS-CSCs. Daunorubicin-induced apoptosis was found to be associated with p53 accumulation, activation of the caspase cascade, and oligonucleosomal DNA fragmentation. Treatment with the caspase inhibitor abolished daunorubicin-induced DNA fragmentation and was therefore considered to act downstream of caspase activation. This was also suppressed by treatment with a Ca^2+^-specific chelator, which suggested that CAD endonuclease does not contribute. Moreover, no obvious ICAD reduction/degradation was detected.

**Conclusion:** Daunorubicin effectively eliminated CSCs, which are dependent on the p53/caspase signaling cascade. The current findings provided the basis for further studies on CSC-targeted drugs for the development of cancer treatment strategies.

## Introduction

Cancer stem cells (CSCs) are considered to be responsible for various pathological phenomena in cancer, such as tumor initiation, growth, tumor vascularization, recurrence, and metastasis^[1,2]^. Multiple studies have consistently reported that the higher resistance capacity of CSCs against chemo- and/or radio-therapies compared to those of non-stem cancer cells significantly contributes to CSC survival after treatment. The mechanisms underlying CSC-mediated resistance have been explained from various points of view, such as the expression of multi drug antiporters and slower cell cycle progression of CSC (quiescent/dormant states) in tumors^[3]^. Recent studies have revealed that the tumor microenvironment also plays important roles in the development of resistance to therapy^[4,5]^. Thus, further understanding not only the nature of CSC itself but also the relationship between CSCs and their microenvironment is required for the development of treatment strategies that can effectively eliminate CSCs from tumors^[1,6]^.

Given that CSC properties are regulated by the CSC niche, drug screening should be carried out in the presence of CSC niches. However, the maintenance and amplification of CSCs and the reconstruction of their niche *in vitro* are challenging when conducting large-scale drug screening^[7]^. Recently, we have established mouse induced pluripotent stem cells (miPSCs) that possess self-renewal and differentiation capacities, as well as malignant tumorigenicity, which is in strong agreement with the definition of CSC^[8-10]^. These miPSCs-derived CSCs (miPS-CSCs) could be maintained *in vitro* under conditions that allow spontaneous differentiation. The miPS-LLCcm cell line (miPSC-CSC differentiated with Lewis lung carcinoma conditioned medium) has been demonstrated to exhibit self-renewal ability by treatment with factors secreted not only autocrinally from the CSCs, but also paracrinally from the differentiated progeny of miPS-LLCcm cells^[11]^. Notch signaling in self-renewing CSCs of miPS-LLCcm is likely to be activated by factor(s) secreted from the endothelial-like cells that were differentiated from CSCs of miPS-LLCcm. Additionally, we reported that culturing miPS-LLCcm cells without the differentiated population influenced the endothelial differentiation capacity of CSCs, thereby suggesting that the signal(s) from the endothelial cells contribute to endothelial differentiation of CSCs^[11,12]^. Thus, we concluded that the generated miPS-CSCs established their niche *in vitro* by autonomously sustaining self-renewal and differentiation, consistent with the endothelial/perivascular niche of CSCs^[13-17]^. miPS-CSCs could serve as an advanced model for the development of anti-CSC drugs.

In the present study, we performed drug screening to identify effective drugs against CSCs using miPS-CSCs, the miPS-derived models of CSCs. In addition we examined the effectiveness of the drug candidates, especially the apoptotic pathway induced by the anthracycline daunorubicin in miPS-LLCcm.

## Methods

### Cell culture

MiPSCs (iPS-MEF-Ng-20D-17)^[18]^ were obtained from the RIKEN Cell Bank and maintained on mitomycin C-treated MEF on gelatin-coated dishes in miPS medium containing Dulbecco’s modified eagle’s medium-high glucose (DMEM, Sigma, MO), 15% FBS (Gibco, NY), 0.1 mmol/L non-essential amino acids (Gibco, NY), 2 mmol/L L-glutamine (Nacalai Tesque, Japan), 0.1 mmol/L 2-mercaptoethanol (Nacalai Tesque, Japan), 50 U/mL penicillin (Nacalai Tesque, Japan), 50 µg/mL streptomycin (Nacalai Tesque, Japan), and 1000 U/mL Leukemia inhibitory factor (LIF, Millipore, MA). The miPS-CSCs were maintained in miPS media without LIF on gelatin-coated dishes^[8,9]^. Mouse Lewis lung carcinoma (LLC) cells, mouse normal fibroblast BALB/c 3T3 A31-714 C4 cells, and human cervical cancer Hela cells were maintained in DMEM containing 10% FBS, 100 U/mL penicillin, and 100 U/mL streptomycin. All cells were cultured at 37 °C with 5% CO_2_ under humidified conditions. The mouse leukemia cell line L1210 (JCRB cell bank, Osaka, Japan) was maintained in RPMI1640 containing 10% FBS, 2 mmol/L L-glutamine, 100 U/mL penicillin, and 100 µg/mL streptomycin.

To prepare the CM from miPS-CSCs, cells were cultured until reaching 80% confluence, after which the culture medium was replaced with serum-free culture medium containing Insulin-transferrin-delenium-x (ITS-X, Life Technologies). CM was collected at 20 h after medium replacement, centrifuged, and then filtered using a 0.45-µm filter (Millipore). For selection of a stem cell-like population in miPS-CSCs, cells were cultured in the medium containing CM of each miPS-CSC (1:1 to DMEM), 0.3 µg/mL for miPS-B16cm cells, or 1 µg/mL for the rest of miPS-CSCs of puromycin for 7 days with one or two passages. The medium was replaced every 24 h during the selection.

After verifying tumorigenicity by injection into the mice biaxillary, the miPS-LLCcm primary cells were obtained from the primary tumors, and the miPS-LLCcm LMT cells were obtained from the resulting lung metastatic tumors.

### Drug screening and evaluation of concentration-dependent cytotoxicity of daunorubicin

All test compounds were provided as Screening Committee of Anticancer Drugs(SCADs) Inhibitor Kit 1 (ver. 2.4) and 2 (ver. 2.0) from SCADs supported by Grant-in-Aid for Scientific Research on Innovative Areas, Scientific Support Programs for Cancer Research from the Ministry of Education, Culture, Sports, Science and Technology, Japan.

Stem cell populations of miPS-CSCs selected in the presence of puromycin were seeded in gelatin-coated 96-well plates at a density of 1 × 10^4^ cells/well in medium containing CMs and DMEM at a 1:1 ratio. After 24 h, test compounds were added at a concentration of 1 μmol/L. Cell viabilities at 48 h were determined as follows. After incubation, the wells were added with 3-(4,5-di-methylthiazol-2-yl)-2,5-diphenyltetrazolium bromide, yellow tetrazole (MTT, Sigma-Aldrich) to a final concentration of 0.6 mg/mL, and the plate was incubated for 4 h. Formed formazan crystals were dissolved in 10% (w/v) SDS with 0.02 N HCl and incubated overnight. Finally, the absorbance of each well was measured at 570 nm using an MTP-800AFC microplate reader (Corona, Japan).

For the analysis of dose-dependent cytotoxicity of daunorubicin against various cells, cells were seeded in 96-well plates. Daunorubicin was added at various concentrations after incubation for 24 h. Viable cells were evaluated by MTT assay as described above. The experiment was independently repeated three times. The concentrations at which cell growth was inhibited by 50% (IC_50_) were determined based on the survival curve obtained by MTT assay.

For the preparation of DNA, RNA, and proteins and for FACS analysis, cells were seeded in a 60-mm dish at a density of 7 × 10^5^ cells/dish. After 24 h, cells were treated with daunorubicin at varying incubation periods.

### Analysis of apoptotic features

To analyze the production of the apoptotic DNA ladder, cells were lysed with a lysis buffer (10 mmol/L Tris-HCl, pH 7.4, 5 mmol/L EDTA, and 0.2% Triton X-100), vortexed, and incubated on ice for 30 min. The cells were then centrifuged (17,400 × *g*, 30 min) to remove precipitated intact genomic DNAs. Supernatants were collected, and fragmented DNA was precipitated with ethanol. Precipitants were re-dissolved in extraction buffer containing 10 mmol/L Tris-HCl (pH 7.4) and 5 mmol/L EDTA and subsequently treated with RNase A for 5 h at 37 °C. Afterwards, cells were added with 1/10 volume of buffer containing 100 mmol/L Tris-HCl (pH 8.0), 100 mmol/L EDTA, and 250 mmol/L NaCl, after which the samples were treated with Proteinase K at 65 °C overnight. DNA fragments were extracted with phenol/chloroform/isoamyl alcohol (25:24:1), precipitated with ethanol, and analyzed by 1.5% or 2% agarose gel electrophoresis.

Annexin V Apoptosis detection kit APC (Affymetrix) was used for the detection of phosphatidylserine as an apoptosis marker. Cells were treated with 100 nmol/L daunorubicin for 3 h, harvested, and stained with annexin V-APC according to the manufacturer’s protocol. Stained cells were analyzed by flow cytometry using BD Accuri C6 (BD Biosciences) equipped with the FlowJo software (Tree Star, Inc., CA).

### RNA preparation, cDNA synthesis, and quantitative PCR

Total RNA was extracted from the cells treated with 100 nmol/L daunorubicin for varying incubations periods using Trizol reagent (Ambion). After DNase I (Takara, Japan) treatment, RNA was re-purified using Trizol reagent. cDNA was synthesized from 3 μg of RNA using SuperScript III First Strand Synthesis Kit (Invitrogen) using oligo-dT primers. Quantitative real-time PCR was performed on a Light Cycler 480 (Roche) instrument and SYBR Green I Master Mix (Roche) according to the manufacturer’s instructions. The following primers were used for PCR. Noxa, 5’-GAGTGCACCGGACATAACTG-3’ and 5’-CTCGTCCTTCAAGTCTGCTG-3’; Bax, 5’-TAGCAAACTGGTGCTCAAGG-3’ and 5’-TCTTGGATCCAGACAAGCAG-3’; Puma, 5’-GTACGAGCGGCGGAGACAAG-3’ and 5’-GCACCTAGTTGGGCTCCATTTCTG-3’; p21, 5’-GCCCGAGAACGGTGGAACTT-3’ and 5’-GACAAGGCCACGTGGTCCTC-3’; Mdm2, 5’-CTAGCTTCTCCCTGAATGCC-3’ and 5’-TTGCACACGTGAAACATGAC-3’; Nanog, 5’-AGGGTCTGCTACTGAGATGCTCTG-3’ and 5’-CAACCACTGGTTTTTCTGCCACCG; Gapdh, 5’-AACGGCACAGTCAAGGCCGA-3’ and 5’-ACCCTTTTGGCTCCACCCTT-3’. The expression levels of Noxa, Bax, Puma, p21, Mdm2, and Nanog genes were normalized against Gapdh expression levels.

### Protein preparation and western blotting

Whole cell lysates were prepared in 50 mmol/L Tris-HCl, pH 7.5 containing 150 mmol/L NaCl, 5 mmol/L EDTA, and 0.5% w/v Triton X-100. Protein concentrations were determined by Bio-Rad Protein Assay (Bio Rad, VA) using normal IgG (Bio Rad) as a standard. Cells that detached from dishes after daunorubicin treatments were collected by centrifugation at 5000 × *g* at 4 °C for 5 min, washed twice with ice cold PBS, and lysed as described above. Cell Fractionation Kit-Standard (ab109719, Abcam, UK) was used to obtain the mitochondrial, nuclear, and cytoplasmic protein fractions according to the manufacturer’s protocol. Afterwards, 15 or 30 μg of protein of whole cell lysate, and the equivalent volume of the cell fractionation were analyzed by SDS-PAGE and electrically transferred on PVDF membranes. The primary and secondary antibodies were used to detect the proteins on the membrane. The following primary antibodies were used for western blotting: anti-p53 antibody in 1:1000 dilution (#2524, Cell Signaling Technology, MA), anti-β-tubulin antibody in 1:600 dilution (#2146, Cell Signaling Technology, MA), anti-Histone H3 antibody in 1:2000 dilution (ab1791, Abcam, UK), anti-Nanog antibody in 1:1000 dilution (ab80892, Abcam, UK), anti-β-actin antibody in 1:1000 dilution (#4970, Cell Signaling Technology, MA), anti-caspase-3 antibody in 1:1000 dilution (#9665, Cell Signaling Technology, MA), anti-cleaved caspase-3 antibody in 1:500 dilution (#9664, Cell signaling Technology, MA), anti-caspase-7 in 1:1000 dilution (#9492, Cell Signaling Technology, MA), anti-caspase-9 antibody in 1:1000 (#9504, Cell Signaling Technology, MA), anti-ICAD antibody in 1:1000 dilution (ab108521, Abcam, UK), anti-cleaved PARP-1 antibody in 1:1000 dilution (#9544, Cell Signaling Technology, MA) and anti-EndoG antibody in 1:1000 (Cell Signaling Technology, MA) were employed. As the secondary antibodies, HRP-conjugated anti-rabbit IgG antibody in 1:10000 dilution (#7074, Cell Signaling Technology, MA), and HRP-conjugated anti-mouse IgG antibody in 1:5000 dilution (sc-2005, Santa Cruz Biotechnology, CA). Immunoreactivity signals were developed using an ECL kit (GE Healthcare, CA) and detected using Light Capture II (ATTO, Japan).

## Results

### Identification of drugs effective against miPS-CSCs

**Figure 1:**
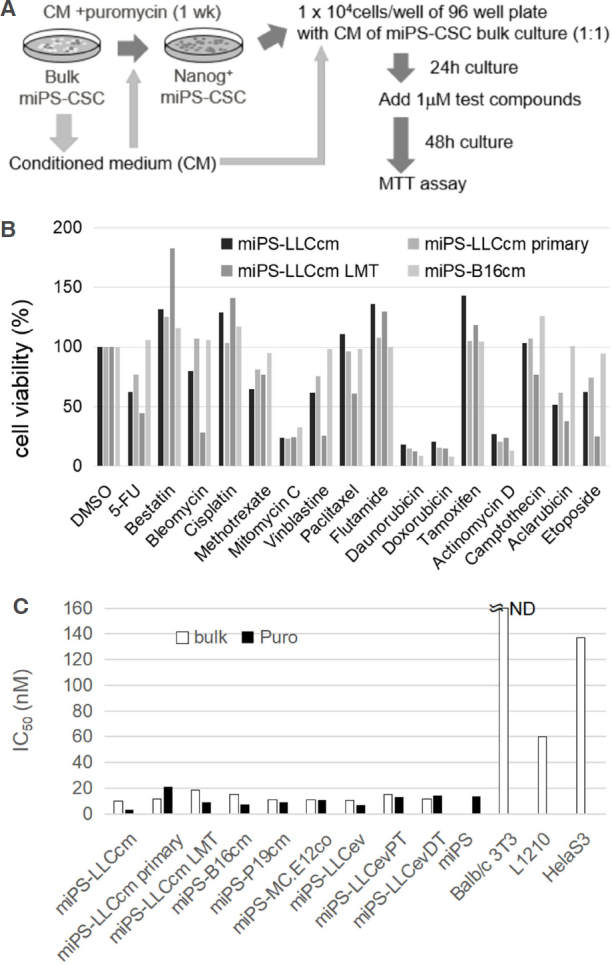
Design of a drug screening system against miPSCs-derived CSCs (miPS-CSCs). A: Schematic representation of drug screening against miPS-CSCs. Stem cell populations of miPS-CSCs were concentrated in the presence of puromycin and CM of bulk culture. Drug screening was performed under treatment with CMs. Each test compound was added at a concentration of 1 μmol/L. Cell viability was determined by MTT assay after 48 h of treatment; B: representative results of screening with compounds categorized as anticancer drugs (*n* = 2). The miPS-LLCcm, miPS-LLCcm primary, miPS-LLCcm LMT, and miPS-B16cm cell lines were used for the screening. Relative cell viabilities were presented as normalized values against DMSO control; C: IC50 values of daunorubicin against various miPS-LLCcm were determined. BALB/c 3T3 cells, L1210s cells, Hela cells, and miPSCs were additionally tested for comparison

The miPS-LLCcm cells are cancer stem-like cells that are spontaneously generated from miPSCs cultured in the presence of CM of mouse Lewis lung cancer cells^[8]^. Given that the parental miPSCs express GFP and puromycin-resistant gene under the control of the Nanog promoter, GFP-expressing Nanog-positive stem cell populations of miPS-LLCcm cells could be concentrated when cultured in the presence of puromycin^[10,11,18]^. Based on our previous findings, puromycin selection of stem cells was carried out in the presence of CM prepared from the bulk culture of miPS-LLCcm cells to fully mimic the niche of miPS-LLCcm cells, which comprise both differentiated and undifferentiated cell populations^[11]^. Furthermore, we designed the drug screening system against puromycin-selected CSCs in medium containing CM from bulk culture of miPS-CSC [Figure 1A]. The cell viabilities under different conditions were determined as described in “Materials and Methods”. In the present study, 190 different chemical compounds categorized as anticancer drugs or cellular signal inhibitors were evaluated against four miPS-CSC cell lines. The analysis identified several compounds that exhibited significant growth inhibitory or cytotoxic effects on miPS-CSCs [Table 1]. The results obtained using several anticancer drugs are summarized in Figure 1B. The miPS-CSCs assessed in the present study were highly sensitive to doxorubicin, daunorubicin, mitomycin C, and actinomycin D, thereby implying that DNA damage and inhibition of DNA replication and/or transcription are effective against miPS-CSCs. However, cisplatin, which can also interfere DNA replication, was not effective at the concentration of 1 μmol/L. The cytotoxic effects of daunorubicin were further analyzed [Figure 1C]. The IC_50_ values of daunorubicin against several different miPS-CSCs in both bulk cell culture and stem cell populations selected by puromycin ranged from 5 to 20 nmol/L. The obtained IC_50_ values against L1210 cells was 60 nmol/L, while that against Hela cells was 137 nmol/L; daunorubicin was not effective on BALB/c 3T3 cells, indicating that daunorubicin effectively inhibits the growth of both undifferentiated and differentiated cells of miPS-CSCs.

### Daunorubicin-induced apoptotic cell death of miPS-LLCcm cells

**Figure 2:**
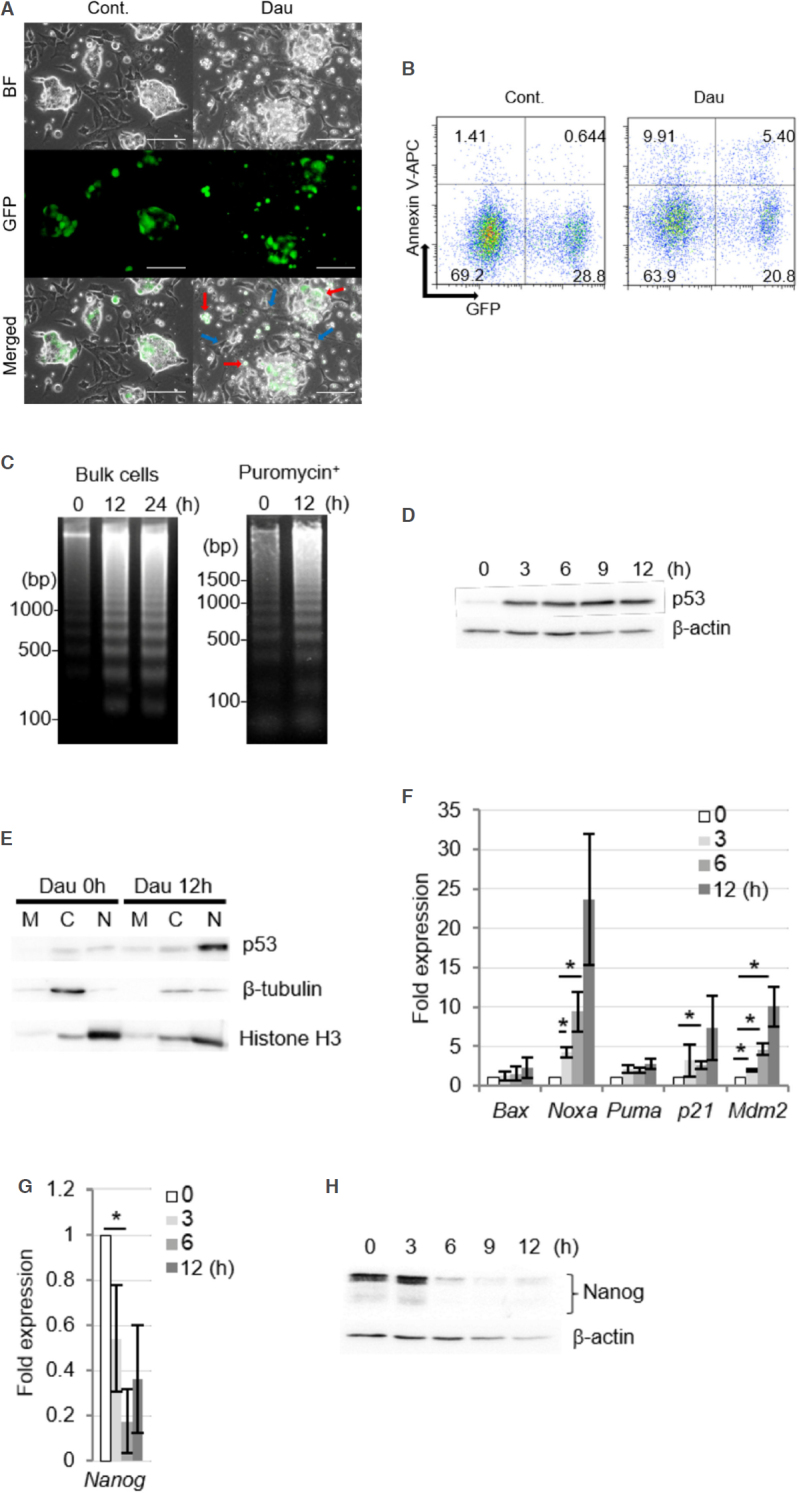
Daunorubicin induces apoptotic cell death in miPS-LLCcm cells with DNA fractionation. A: Morphology of miPS-LLCcm cells undergoing cell death. Cells were treated with daunorubicin at a concentration of 100 nmol/L. Images were captured after 12 h of treatment. Bar: 50 mm. Red arrows show dying miPS-LLCcm cells, and blue arrows show non-apoptotic cells; B: detection of apoptotic cells by annexin-V staining. miPS-LLCcm were treated with 100 nmol/L daunorubicin for 3 h, harvested, and stained with annexin-V APC. Flow cytometry was performed twice, and the representative results are shown; C: detection of oligonucleosomal DNA ladder in bulk of miPS-LLCcm and in stem cells. DNA was prepared from miPS-LLCcm bulk (left) or puromycin-treated (right) cells subsequently treated with 100 nmol/L daunorubicin for the indicated periods and subjected to agarose gel electrophoresis. Nuclear accumulation of p53 protein following daunorubicin treatment; D: miPS-LLCcm cells were treated with daunorubicin for the indicated periods. Whole cell lysates prepared and analyzed by western blotting using p53 antibody. β-actin levels were used as internal control; E: nuclear accumulation of p53 proteins at the indicated periods was fractionated and subjected to western blotting. M: Mitochondrial, C: cytoplasmic, and N: nuclear. β-tubulin and Histone H3 were detected for fractionation markers; F: expression of p53-regulated genes in daunorubicin-treated miPS-LLCcm cells. RT-qPCR was performed using cDNA prepared from cells treated with 100 nmol/L daunorubicin for the indicated periods (*n* = 3); G: suppression of Nanog gene expression was confirmed by RT-qPCR. Error bars in Figure 2E and F are shown as SD; H: reduction in Nanog protein levels after daunorubicin treatment. Whole-cell lysates prepared from daunorubicin-treated cells were subjected to western blot analysis

Daunorubicin and doxorubicin are topoisomerase II inhibitors that cause DNA strand breaks and induce DNA damage responses and apoptotic cell death^[19]^. In the present study, we assessed the death of miPS-LLCcm cells following treatment with 100 nmol/L daunorubicin. Small vesicles that appeared similar to apoptotic bodies were frequently observed on GFP-positive cells during daunorubicin treatment [Figure 2A]. After 3-h exposure to daunorubicin, significant proportions of annexin V-stained cells were detected for both GFP-positive and -negative cells [Figure 2B]. DNA fragment isolation could remove intact genomic DNA by centrifugation immediately after cell lysis, which could significantly increase the sensitivity to allow the detection of fragmented DNA. Thus, the amounts of intact DNA in healthy cells were very low in the background such that apoptotic DNA fragmentation was strongly evident [Supplementary Figure 1]. The typical oligonucleosomal DNA ladder was clearly observed after 12 h of treatment with danuorubicin in both the bulk cells and stem cells selected by puromycin [Figure 2C]. Taken together, the above results indicated that miPS-LLCcm cells, including the populations comprising both GFP-negative differentiated cells and GFP-positive stem cells, underwent apoptotic cell death.

Next, we assessed the involvement of the p53 pathway in the apoptosis of miPS-LLCcm cells. Significant accumulation of p53 proteins was observed upon treatment with daunorubicin [Figure 2D] and was also observed in parental miPS and the primary cells established from the tumor formed by transplantation of the miPS-LLCcm cells in a nude mouse^[8]^. The p53 proteins were concentrated in the nuclear fraction after daunorubicin treatment [Figure 2E]. The p53 transcription factor induces the expression of several genes involved in DNA damage response, such as apoptosis and cell cycle regulation. In the miPS-LLCcm cells, the expression of apoptotic factors, such as Noxa, Bax, and Puma, as well as the CDK inhibitor p21, and Mdm2, a negative regulator of p53, were found to be upregulated upon daunorubicin treatment [Figure 2F]. The induction of Noxa expression was especially prominent, indicating the involvement of the Noxa-dependent mitochondrial apoptosis pathway^[20]^. In addition to the upregulated expression of certain target genes, p53 represses the expression of various genes. For instance, p53 has been demonstrated to repress Nanog expression in mouse embryonic stem cells by binding to its promoter after DNA damage^[21,22]^. Nanog expression in miPS-LLCcm cells was reduced within 3 h after daunorubicin treatment [Figure 2G]. In addition, Nanog protein levels were dramatically reduced after of 3 to 6 h of treatment [Figure 2H]. Furthermore, given that Nanog was predominantly expressed in stem cells, the above findings suggested that the p53 pathway is activated in the stem cell population of miPS-LLCcm cells.

### ICAD/CAD-independent apoptosis in miPS-LLCcm cells

**Figure 3:**
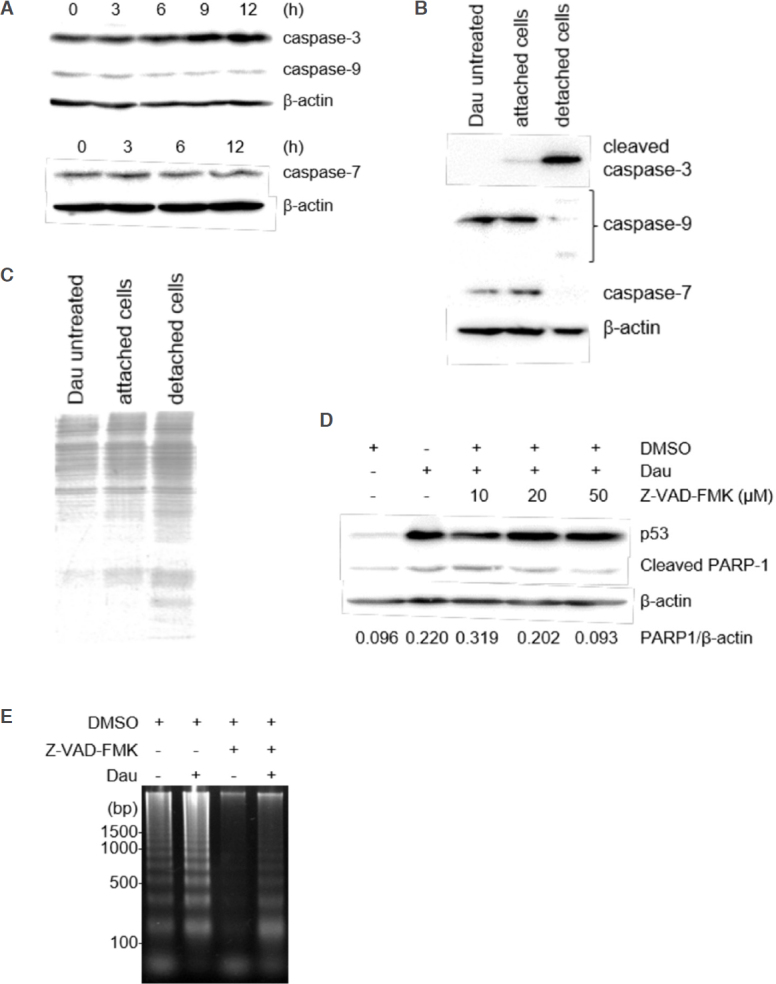
Involvement of caspases in the apoptosis of miPS-LLCcm cells. A: Expression levels of procaspases during daunorubicin-induced cell death of miPS-LLCcm cells. miPS-LLCcm were treated with 100 nmol/L daunorubicin for the indicated periods. Whole cell lysates were prepared and analyzed by western blotting; B: detection of processed caspases in cells attached or detached to the dish. After daunorubicin treatment, floating cells were concentrated by centrifugation, and whole-cell lysates were prepared. Cleaved caspase-3, procaspase-9, and procaspase-7 levels were detected; C: confirmation of whole proteins in detached cells. Cell lysates were subjected to SDS-PAGE and stained with CBB; D: suppression of cleavage of PARP-1 by Z-VAD-FMK. Cells were treated with 100 nmol/L daunorubicin in the presence of the pan-caspase inhibitor, Z-VAD-FMK. Cleaved PARP-1 was analyzed by western blotting; E: suppression of DNA fragmentation by Z-VAD-FMK. DNA fragmentation in cells treated with daunorubicin in the presence of 50 μmol/L of Z-VAD-FMK were analyzed

Given that the apoptotic DNA ladder was observed after 12 h of daunorubicin treatment [Figure 2C], we next assessed the activation of caspases-ICAD/CAD pathway during cell death of miPS-LLCcm cells. We investigated several caspases and their substrates by western blotting [Figure 3A]. However, we could not detect significant cleavage or reduction of full-length caspases [both the executioner (caspase-3 and -7) and the initiator caspase (caspase-9)] in the cells attached on dish after daunorubicin treatment. Although an antibody specific to the cleaved caspase-9 failed to detect the protein, trace levels of cleaved caspase-3 were observed (data not shown). Therefore, the cells in medium that detached from their dish after programmed cell death were analyzed, and the activation of caspase 3 and other caspases could be detected. Results showed that caspase-3 activation, cleavage of caspase-9, and reduced levels of full-length caspase-7 were significantly detected in detached cells, demonstrating that the activation of caspase-cascade is involved in the daunorubicin-induced apoptotic cell death of miPS-LLCcm cells [Figure 3B]. SDS-PAGE analysis confirmed that overall proteolysis did not occur in these cells at this point [Figure 3C]. Caspase activation was further confirmed by treatment of the cells with an inhibitor of pan-caspase, Z-VAD-FMK. Daunorubicin treatment increased the levels of cleaved PARP-1, a substrate of caspase-3 and -7, during apoptosis; however, treatment with 50 μmol/L Z-VAD-FMK blocked the accumulation of cleavage of PARP-1. We observed no significant changes in p53 levels in the presence of Z-VAD-FMK, indicating that apoptosis induction itself was not inhibited [Figure 3D]. Under this condition, the levels of apoptotic fragmented DNA was significantly reduced [Figure 3E], thereby indicating that the activation of the caspase cascade was induced but not essential for daunorubicin-induced apoptotic cell death of miPS-LLCcm cells.

**Figure 4:**
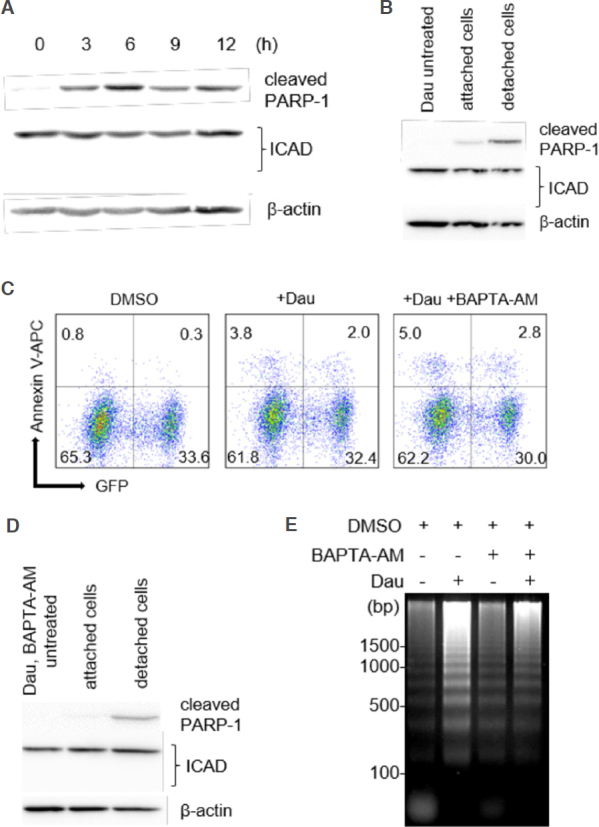
Potential involvement of Ca^2+^/Mg^2+^-dependent endonuclease in DNA fragmentation. A: We observed no obvious reduction in full-length ICAD levels during apoptosis of miPS-LLCcm. miPS-LLCcm cells were treated with 100 nmol/L of daunorubicin for the indicated periods and analyzed by western blotting analyses; B: detection of processed caspases in attached or floating cells; C: apoptotic cells in the presence of BAPTA-AM were detected by annexin V staining. Cells were pretreated with 10 μmol/L of BAPTA-AM for 1 h, treated with 100 nmol/L daunorubicin, and analyzed by FACS; D: Ca^2+^ chelator did not affect caspase levels during apoptosis of miPS-LLCcm cells; E: suppression of DNA fragmentation by treatment with a Ca^2+^ chelator. DNA fragmentation in the cells treated with daunorubicin in the presence of 10 μmol/L of BAPTA-AM was analyzed

We next assessed the degradation of ICAD, another caspase-3 substrate that is required for the release of an endonuclease CAD, which is responsible for the formation of the apoptotic DNA ladder^[23-25]^. Whereas the cleaved PARP-1 levels were readily detectable and significantly increased within the first 3 h of daunorubicin treatment, the full-length ICAD levels remained stable throughout the treatment period, and no cleaved/degraded ICAD fragments were observed [Figure 4A]. We observed no reduction in ICAD degradation even in the concentrated detached cells [Figure 4B].

Ca^2+^/Mg^2+^-dependent endonucleases have been reported to contribute to apoptotic DNA fragmentation^[26-31]^, whereas Ca^2+^ is not required for CAD activity^[32]^. Next, we assessed the effect of BAPTA-AM, a Ca^2+^-specific cell chelator, on the formation of apoptotic DNA ladder. Apoptosis induced by daunorubicin was not inhibited in the presence of 10 μmol/L BAPTA-AM based on the cleavage of both caspase-3 and PARP-1 together with positive staining of annexin V-APC [Figure 4C]; however, constitutive levels of full-length of ICAD were detected [Figure 4D]. Interestingly, DNA fragmentation was suppressed under this condition [Figure 4E], suggesting the involvement of Ca^2+^/Mg^2+^-dependent endonucleases during daunorubicin-induced apoptotic cell death of miPS-LLCcm cells.

**Figure 5:**
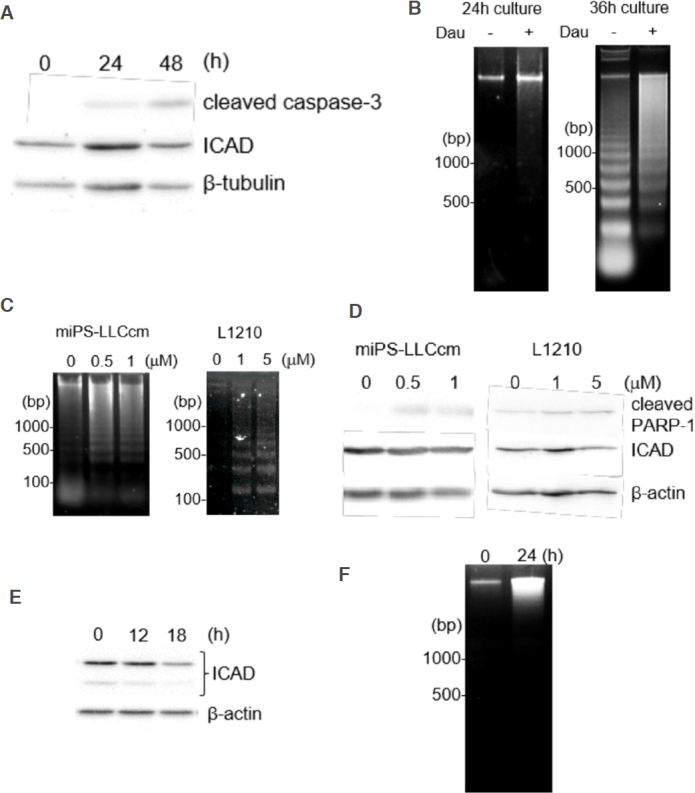
Comparison of apoptotic features among L1210, miPS-LLCcm, and Hela cells. A: Mouse leukemia L1210 cells were treated with 1 μmol/L daunorubicin for the indicated periods. Protein levels of activated caspase-3 and its substrate ICAD were determined by western blotting; B: DNA fragmentation in L1210 cells was analyzed. Cells were treated with daunorubicin for 24 or 36 h; C: DNA fragmentation in miPS-LLCcm and L1210 cells treated with staurosporine. Cells were treated with various concentrations of staurosporine for 3 h (miPS-LLCcm) or 12 h (L1210), after which DNA fragmentation was observed; D: Western blot analysis of PARP-1 and ICAD levels in staurosporine-treated cells (3 h for miPS-LLCcm and 12 h for L1210); E: ICAD levels in Hela cells treated with 1 μmol/L daunorubicin for the indicated periods were analyzed by western blotting; F: DNA fragmentation in Hela cells treated with 1 μmol/L daunorubicin

Daunorubicin is clinically used for the treatment of acute myeloid leukemia. To evaluate the cytotoxic effects of daunorubicin, we assessed the apoptotic features in mouse leukemia cell line L1210 cells treated with daunorubicin. Daunorubicin activated the p53 and caspase cascades in L1210 cells. However, ICAD degradation was not observed in L1210 cells [Figure 5A]. Interestingly, DNA fragmentation in L1210 cells was different from that observed in miPS-LLCcm cells. In fact, the oligonucleosomal DNA ladder was detected even when L1210 cells were cultured for more than 36 h without daunorubicin, thereby indicating cell death during overgrowth; therefore, this condition should be treated as the background condition. In the presence of daunorubicin, the smearing DNA fragmentation was prominent after 36 h of treatment, while the oligonucleosomal DNA ladder was not detected within 24 h [Figure 5B]. For ICAD degradation, we assessed the apoptosis induced by staurosporine in both miPS-LLCcm and L1210 cells. Although the two cell lines showed different sensitivities to staurosporine, oligonucleosomal DNA fragmentation was detected in both cells [Figure 5C]. Under this condition, ICAD levels in miPS-LLCcm cells remained stable, whereas ICAD levels in L1210 cells were reduced [Figure 5D].

We further tested ICAD degradation and DNA fragmentation in human Hela cells. After treatment of Hela cells with daunorubicin, we observed time-dependent reduction of full-length ICAD, indicating CAD activation [Figure 5E]. However, oligonucleosomal DNA fragmentation was not detected [Figure 5F]. Taken together, our results suggested that daunorubicin affects ICAD degradation depending on the cell type and that CAD contributes little to the formation of oligonucleosomal DNA ladder during apoptosis of miPS-LLCcm.

## Discussion

The behavior of CSCs is considered to be regulated in the niche by mutual communication between CSCs, their progenies, and adjacent normal cells. Recent studies have shown that endothelial cells play important roles in regulating CSC properties as components of the CSC niche^[11,13-17]^. Various studies, including our previous studies, indicated that tumor endothelial cells are the progenies of CSCs^[9-11,33-35]^. Regarding therapy resistance, studies have shown that both senescent and non-senescent tumor microvascular endothelial cells that are resistant to radiotherapy and chemotherapy could still contribute to the growth of CSCs in glioblastoma multiform^[5]^. Considering the significant role of the CSC niche, *in vitro* screening of cancer drugs should be performed under the conditions that mimic the CSC niche. Our miPS-CSCs have been confirmed to exhibit the potential to create an endothelial niche-like environment *in vitro*^[10,11]^, in which soluble factors, including Notch ligands, were found to promote self-renewal and differentiation of CSCs^[11]^. Thus, we performed the experiments using purified miPS-CSCs in the presence of CM obtained from bulk miPS-CSCs.

Various anticancer drugs induce apoptosis in target cells. Processes related to the intrinsic apoptotic pathways, such as DNA damage, are generally mediated by the p53/mitochondria/caspase cascade. To release caspase-activated DNase (CAD/DFF40), activated executor caspases, such as caspase-3, 6, and 7, cleave the inhibitor of CAD (ICAD/DFF45). CAD is a major endonuclease that contributes to the formation of oligonucleosomal DNA fragmentation during apoptosis^[23-25,36]^. Chemo- or radio-therapies that cause DNA lesions are expected to activate this pathway to eliminate target cells. However, nearly half of malignant cancers harbor mutations in the p53 gene that are believed to be a critical for developing malignancy. On the other hand, the rest of cancers retain wild-type p53 gene and are thought to be inactivate the p53 pathway through different mechanisms. In the case of the teratocarcinoma cell line NTera2, the cells expresses wild-type p53, but the transcriptional activity is suppressed by methylation of lysine residues^[37]^. Interestingly, teratocarcinoma cells undergo apoptosis upon DNA damage, suggesting that the suppression mechanism is removable^[38]^. Thus, (re)activation of p53 could be effective in eliminating tumors in various types of cancers. Similarly, normal embryonic stem cell express the p53 gene, and p53-mediated cell death or differentiation can be induced^[21,26]^. Therefore, p53 is also speculated to regulate CSC properties, and the strategies for activation of the p53 pathway by stimuli, such as DNA damage, have been adopted to eliminate CSCs^[39]^.

Daunorubicin was found to induce oligonucleosomal DNA fragmentation, a typical feature of apoptotic cell death. CAD/DFF40 is the major endonuclease responsible for this DNA fragmentation^[23]^. Under non-apoptotic conditions, CAD is bound to the inhibitor ICAD/DFF45, thereby repressing CAD activity. Once apoptosis triggered, executor caspase-3, 6, or 7 cleaves ICAD to release CAD, after which the endonuclease activity leads to DNA fragmentation. However, in the present study, ICAD cleavage was not observed despite the apparent activation of the p53-caspase pathway, thereby resulting in PARP-1 cleavage. The observed discrepancy can be explained by the more efficient cleavage of PARP by caspase-7 than ICAD, while the catalytic efficiencies of caspase-3 for ICAD and PARP were comparable^[25]^. The above findings showed that both caspases were activated by daunorubicin treatment in miPS-LLCcm cells, indicating that dominant PARP-1 cleaved by caspase-7 is feasible. Post-translational modification of ICAD is a likely mechanism behind resistance to ICAD degradation. Recently, *O*-linked beta-*N*-acetylglucosamine (O-GlcNAc) modification of proteins has been demonstrated to regulate apoptosis under conditions of various cellular stresses^[40,41]^. ICAD could be modified with O-GlcNAc, and this modification protects cleavage by caspases in T cells^[42]^. Furthermore, O-GlcNAc modification of proteins regulates the differentiation of ES cells^[43]^. Similarly, the oligonucleosomal DNA fragmentation without ICAD degradation was described during apoptosis of immature B-cell lines induced by B-cell receptor ligation^[31]^. Moreover, treatment with DNA topoisomerase II inhibitors, both daunorubicin and doxorubicin, has been reported to promote histone eviction and attenuate the DNA damage response^[44]^. Although epigenetic analysis was not conducted in the present study, treatment with daunorubicin and doxorubicin could be expected to induce DNA fragmentation via that mechanisms.

While Ca^2+^-independent CAD activity is not likely to be responsible for the DNA fragmentation observed in miPS-LLCcm cells duringir daunorubicin-induced apoptosis [Figure 5E]^[32]^. Ca^2+^/Mg^2+^-dependent endonucleases, such as EndoG, are known to contribute to oligonucleosomal DNA fragmentation. EndoG is a mitochondrial endonuclease that translocates to the nucleus during apoptosis^[27]^. Cell death of mouse embryonic stem cells induced by etoposide, an inhibitor of topoisomerase II, was found to be EndoG-dependent^[26]^ and was proposed as a novel mechanism behind programmed cell death, given that significant activation of caspases was not observed. The contributions of cathepsins for proteolytic observations and EndoG for DNA fragmentation were suspected in this programed cell death. By contrast, our results suggested the involvement of caspases in the cell death of miPS-LLCcm cells, since the inhibition of caspases by pan-caspase inhibitor, Z-VAD-FMK, attenuated DNA fragmentation [Figure 3E]. Therefore, the involvement of caspases in EndoG-mediated DNA fragmentation remains controversial. EndoG translocation was originally reported to occur in a caspase-independent manner^[27]^, whereas the release of this nuclease from mitochondria was described to require caspase activation^[45]^. Positive feedback loop of caspase-3 was proposed to be mediated by the release of AIF/EndoG^[46]^. However, EndoG is not likely to be involved in daunorubicin-induced apoptosis of miPS-LLCcm cells, given that EndoG translocation from mitochondria to nuclei was not detected in the preliminary experiment [Supplementary Figure 2].

Regulation of apoptosis is complex and dependent on the cellular context. Further investigations using our miPS-CSCs, such as selection of endonuclease in DNA fragmentation in CSCs, could shed light on the mechanisms associated with apoptosis. These analyses can help elucidate the mechanisms underlying drug- and apoptosis-resistance in cancer and CSCs and could lead to the identification of novel therapeutic targets. Taken together, we propose that our miPS-CSCs could be useful as models for the screening of anti-CSC drugs, the investigation of characteristic molecular mechanisms associated with apoptosis in CSCs, and the development of novel strategies for the treatment of CSCs.

CSCs are crucial because they are involved in various pathological features of cancer. Thus, strategies for CSC elimination and detailed understanding of CSCs are required to develop and effective treatment for cancer. We proposed that miPS-CSCs established from miPSCs could be useful as models of CSCs for investigating CSC properties in their niche and the formation of the tumor vasculature^[8-11,47,48]^. In this study, topoisomerase II inhibitor, doxorubicin and daunorubicin, were found to be effective in suppressing the growth of miPS-CSCs *in vitro*. In addition, detailed analyses of cell death mechanisms revealed that daunorubicin-induced apoptosis of miPS-LLCcm cells was caspase-dependent but ICAD/CAD-independent for DNA fragmentation. The endonuclease involved in this cell death appeared to be Ca^2+^/Mg^2+^-dependent.

## Declarations

### Copyright

© The Author(s) 2022.
