# Supplementary material for: Daunorubicin can eliminate iPS-derived cancer stem cells via ICAD/CAD-independent DNA fragmentation
Source: Cancer Drug Resist. 2019 Jun 19;2(2):335–50. doi: 10.20517/cdr.2019.01 (PMC8992628; doi:10.20517/cdr.2019.01)
Supplement: Supplementary file 1 [file cdr-2-335-SupplementaryMaterials.pdf]

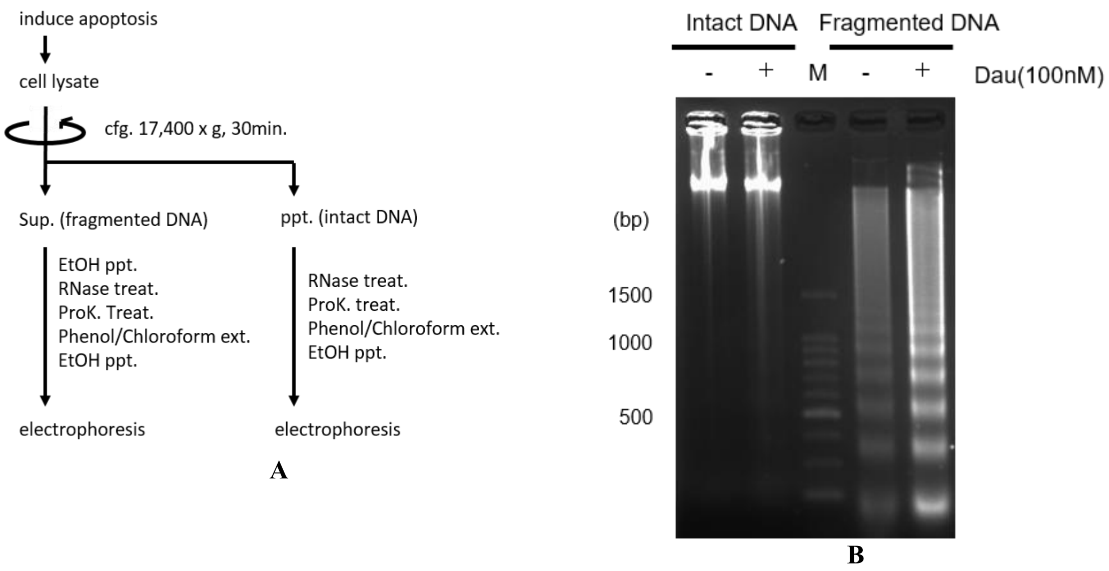

Supplementary Figure 1. Procedure of DNA fragment isolation. (A) A scheme of collecting fragmented DNA. Fragmented DNA was obtained by the centrifuge just after the cell lysis. (B) With this method, fragmented DNAs are clearly appeared in the supernatant of the cell lysate.

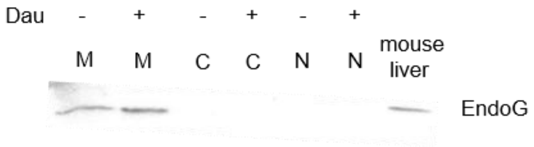

Supplementary Figure 2. EndoG translocation from mitochondria to nuclei was not observed with Daunorubicin treatment. 100nM Daunorubicin was treated to miPS-LLCcm cells for 12 h. M: Mitochondrial, C: cytoplasmic, and N: nuclear. Proteins extracted from mouse liver was used as a positive control.

© The Author(s) 2018. Open Access This article is licensed under a Creative Commons Attribution 4.0 International License (https://creativecommons.org/licenses/by/4.0/), which permits unrestricted use, sharing, adaptation, distribution and reproduction in any medium or format, for any purpose, even commercially, as long as you give appropriate credit to the original author(s) and the source, provide a link to the Creative Commons license, and indicate if changes were made.
